# Influence of epistasis on response to genomic selection using complete sequence data

**DOI:** 10.1186/s12711-017-0340-3

**Published:** 2017-08-25

**Authors:** Natalia S. Forneris, Zulma G. Vitezica, Andres Legarra, Miguel Pérez-Enciso

**Affiliations:** 1 Centre for Research in Agricultural Genomics (CRAG), CSIC-IRTA-UAB-UB Consortium, 08193 Bellaterra, Barcelona, Spain; 2 Departamento de Producción Animal, Facultad de Agronomía, Universidad de Buenos Aires, C1417DSE Buenos Aires, Argentina; 3 GenPhySE, INRA, INPT, ENVT, Université de Toulouse, 31326 Castanet-Tolosan, France; 4 Departament de Ciència Animal i dels Aliments, Universitat Autònoma de Barcelona, 08193 Bellaterra, Barcelona, Spain; 5 ICREA, Passeig de Lluís Companys 23, 08010 Barcelona, Spain

## Abstract

**Background:**

The effect of epistasis on response to selection is a highly debated topic. Here, we investigated the impact of epistasis on response to sequence-based selection via genomic best linear prediction (GBLUP) in a regime of strong non-symmetrical epistasis under divergent selection, using real Drosophila sequence data. We also explored the possible advantage of including epistasis in the evaluation model and/or of knowing the causal mutations.

**Results:**

Response to selection was almost exclusively due to changes in allele frequency at a few loci with a large effect. Response was highly asymmetric (about four phenotypic standard deviations higher for upward than downward selection) due to the highly skewed site frequency spectrum. Epistasis accentuated this asymmetry and affected response to selection by modulating the additive genetic variance, which was sustained for longer under upward selection whereas it eroded rapidly under downward selection. Response to selection was quite insensitive to the evaluation model, especially under an additive scenario. Nevertheless, including epistasis in the model when there was none eventually led to lower accuracies as selection proceeded. Accounting for epistasis in the model, if it existed, was beneficial but only in the medium term. There was not much gain in response if causal mutations were known, compared to using sequence data, which is likely due to strong linkage disequilibrium, high heritability and availability of phenotypes on candidates.

**Conclusions:**

Epistatic interactions affect the response to genomic selection by modulating the additive genetic variance used for selection. Epistasis releases additive variance that may increase response to selection compared to a pure additive genetic action. Furthermore, genomic evaluation models and, in particular, GBLUP are robust, i.e. adding complexity to the model did not modify substantially the response (for a given architecture).

**Electronic supplementary material:**

The online version of this article (doi:10.1186/s12711-017-0340-3) contains supplementary material, which is available to authorized users.

## Background

The relation between the genotype and phenotype of an individual can be extremely complex. Nevertheless, quantitative genetics is able to predict breeding values and response to selection with surprising accuracy based on highly simplified assumptions. Among all potential complexities, epistasis is one of the most widely studied and controversial [1]. In the physiological sense, recently reviewed experimental evidence [2] suggests that functional epistatic gene action is common, and that additivity can be an emergent property of underlying genetic interaction networks. In the statistical sense, functional epistasis makes the statistical additive effects of alleles depend on the current genetic background, and their contribution to the total genetic variance depends on the allele frequencies [3].

The effect of epistasis on response to selection is a highly debated topic, both from the perspective of short-term response and from an evolutionary perspective [4]. Although some authors claim that its effect on the long term may be substantial, others argue that interaction effects contribute very little to the total genetic variance of a population, and consequently to its short-term response, because most of the variance is additive [5]. When epistasis is present, the level of additive variance can be sustained or even increased compared to that expected under a strict additive model and the genetic gain may be sustained for longer [4]. Moreover, if epistasis is not symmetrical, that is, if alleles with a positive marginal effect interact positively on average (or negatively), the rate of evolution will accelerate (or decelerate) [4].

In the presence of functional epistasis, allele frequency drift and changes in frequency of causal alleles due to selection will cause the response to artificial selection from the same base population to differ among replicate lines as well as within the same line over time [2]. Unless interacting loci are identified and co-introgressed, a favorable allele at one locus may be detrimental in a different genetic background. Although it was shown that an additive model may explain a major part of the genetic variance in different datasets [5], this model does not explicitly capture any kind of interaction which may be present in biochemical pathways that connect gene expression with the ultimate target phenotype. Therefore, statistical models that incorporate interactions between loci have been viewed as potentially beneficial for genomic prediction [2, 6–9].

So far, genomic selection (GS) has been mainly performed with manufactured genotyping arrays based on single nucleotide polymorphisms (SNPs), but the drop in sequencing costs should enable GS programs to routinely use genome sequencing instead of genotyping arrays in the near future. Since causative variants are themselves (potentially) included in the sequence data, the accuracy that can be achieved when sequence data is used instead of SNP arrays is expected to be no longer limited by linkage disequilibrium (LD) between SNPs and causal mutations [10]. Nevertheless, a few empirical studies on breeding schemes and recent simulations agree on the fact that full sequence data will probably not make SNP arrays obsolete for predicting genetic merit [11, 12]; yet, a modest increase (~4%) in genomic best linear unbiased prediction (GBLUP) accuracy, compared to SNP arrays, can be expected under some genetic architectures [13–15]. Using sequence and SNP data, VanRaden [16] obtained an average increase of 2.5% in accuracy in US Holstein cattle compared to using SNP data only.

The aim of this study was to quantify the impact of epistasis on the response to GS in an extreme regime of non-symmetrical epistasis in diploid genomes, as well as to study the possible advantage of including non-additive effects in the prediction model. Since large amounts of epistasis have been reported in Drosophila [2], in this study, we mimicked this genome and used real Drosophila sequence data as starting population.

## Methods

We conducted an in silico divergent genomic selection experiment in Drosophila using the sequence based virtual breeding (SBVB) software [15]. SBVB can use real sequence data for founder animals in a simulated population and simulates the genomes and phenotypes of offspring in a very efficient and flexible manner according to specified genetic architectures. Although SBVB is essentially a gene-dropping algorithm, it allows implementing selection by efficiently reading and writing haplotypes (see Additional file 1: Figure S1).

### Data

We downloaded the public SNP data from the 205 inbred lines of the *Drosophila melanogaster* Genetic Reference Panel v2 (DGRP Freeze 2.0, http://dgrp2.gnets.ncsu.edu/ [17]). SNPs from chromosome 4 and indels were removed, resulting in 3,954,651 SNPs that were used for analyses. Chromosome 4 in Drosophila is normally ignored in population genetic studies since it is very small, does not recombine and is mostly heterochromatic. Missing values were imputed with Beagle4 [18]. SBVB allows the specification of variable recombination rates along the genome and between sexes as well as sex chromosomes. We used the genetic map from Flybase (www.flybase.org) and allowed for the fact that no recombination occurs in male Drosophila.

### Genetic architecture

Four hundred causal SNPs, i.e. quantitative trait nucleotides (QTN) were considered in the analysis. We used the 103 SNPs and their estimated additive effects on the phenotype “chill coma recovery time” which are reported by [19] in their supplementary Table 3. For those SNPs that were identified in both sexes (12 out of 103), we used the average between-sex effect size as QTN effect. Otherwise, the specific sex effect was taken as the additive genetic effect in both sexes. In addition, we used 297 randomly chosen SNPs with their additive effects that were simulated by using an exponential distribution with rate parameter equal to 5. The purpose of this was to generate a larger number of loci with smaller effects than those detected by the association study in [19], since the quantitative trait loci (QTL) that are reported as significant are typically those with the largest effects, leading to a marked upward bias [20]. The sign (− or +) of the simulated additive effect sizes was sampled with equal probability.

Genotypic values were simulated according to two extreme architectures.

#### Epistatic architecture

All loci (400 = 103 + 297) were randomly grouped in 200 epistatic pairs. Second-order epistasis followed the complementary model [5], where the genotypic values for the nine possible two-locus genotype combinations are equal to:

$$\begin{array}{*{20}c} {} & {C_{1} C_{1} } & {C_{1} C_{2} } & {C_{2} C_{2} } \\ {B_{1} B_{1} } & z & z & 0 \\ {B_{1} B_{2} } & z & z & 0 \\ {B_{2} B_{2} } & 0 & 0 & 0 \\ \end{array}$$

This architecture assumes equal values for the additive effect of locus *B* and locus *C* and complete dominance (*a* = *d*), with *B*_1_ and *C*_1_ being the dominant alleles. The genotypic value ‘*z*’ was computed as the arithmetic mean of the effects of the two original loci, that is, either the effect reported by [19] or that simulated from the exponential distribution. In this architecture, the double mutation *B*_1_ *C*_1_ yields the same phenotype as either one alone. The genotypic value of an individual was computed as the sum of the two-locus genotypic value at each of the 200 epistatic pairs. We chose the complementary model because it is simple to interpret and allows for substantial non-additive genetic effects (~25% of the genetic variance is non-additive with a ‘U’ shape distribution of allele frequencies) [5].

#### Additive architecture

The genotypic value of an individual was obtained by summing all additive genotypic effects across loci, according to each individual’s genotypes (i.e., *a*, 0 or −*a* for *B*_1_ *B*_1_, *B*_1_ *B*_2_ and *B*_2_ *B*_2_, respectively). Here, the value ‘*a*’ of each locus is equal to the value ‘*z*’ used for the corresponding epistatic pair previously described.

Each individual’s phenotype was calculated from its genotypic value adding an environmental effect taken from a normal distribution with mean 0 and variance $\sigma_{e}^{2}$. In both architectures, $\sigma_{e}^{2}$ was adjusted so that the broad sense heritability ($H^{2}$) was 0.5, before selection started. Narrow sense heritability was approximately 0.25 for the epistatic architecture.

### Evaluation model

Breeding values were predicted using GBLUP [21]. Briefly, GBLUP uses SNPs to build genomic relationship matrices (${\mathbf{G}}$). Four alternative models were used to evaluate individuals.

#### Additive model using sequence data (A-SEQ)

The evaluation model included a mean plus additive values distributed as ${\mathbf{a}}\sim N({\mathbf{0}},{\mathbf{G}}\sigma_{a}^{2} )$, where ${\mathbf{G}}$ was obtained using all SNPs. Computation of ${\mathbf{G}}$ is described below.

#### Additive model using causal SNPs (A-QTN)

As above except that ${\mathbf{G}}$ was obtained by using only causal SNPs (QTN).

#### Full epistatic model using sequence data (E-SEQ)

The evaluation model included a mean, (statistical) additive values, distributed as ${\mathbf{a}}\sim N({\mathbf{0}},{\mathbf{G}}\sigma_{a}^{2} )$, a dominant random deviation ${\mathbf{d}}\sim N({\mathbf{0}},{\mathbf{D}}\sigma_{d}^{2} )$, and an (statistical) epistatic effect distributed as ${\mathbf{h}}\sim N({\mathbf{0}},{\mathbf{G}} \ne {\mathbf{G}}\sigma_{p}^{2} )$, where $\ne$ denotes the Hadamard product [22, 23]; ${\mathbf{G}}$ and ${\mathbf{D}}$ were obtained by using all SNPs. Computation of ${\mathbf{D}}$ is described below.

#### Full epistatic model using causal SNPs (E-QTN)

As above except that ${\mathbf{G}}$ and ${\mathbf{D}}$ were obtained by using only the QTN.

For a given set of markers (all or only causal SNPs), ${\mathbf{G}}$ was obtained from ${\mathbf{MM}}^{\prime } /\sum\nolimits_{{j = 1}}^{k} 2 p_{j} q_{j}$, where the elements of the ${\mathbf{m}}$ vectors for each individual are equal to $- 2p_{j}$, $1 - 2p_{j}$, and $2 - 2p_{j}$ for genotypes *B*_1*j*_ *B*_1*j*_, *B*_1*j*_ *B*_2*j*_ and *B*_2*j*_ *B*_2*j*_, respectively [21], $p_{j}$ is the frequency of allele *B*_1*j*_ for the genotyped individuals of the population, $q_{j} = 1 - p_{j}$, and $k$ is the number of SNPs. ${\mathbf{D}}$ was obtained as in [7]:

$${\mathbf{D}} = \frac{{{\mathbf{M}}_{{\mathbf{d}}} {\mathbf{M}}_{{\mathbf{d}}}^{'} }}{{\mathop \sum \nolimits_{j = 1}^{k} 4p_{j}^{2} q_{j}^{2} }},$$

where the elements of the ${\mathbf{m}}_{d}$ vectors for each individual are equal to $- 2p_{j}^{2}$, $2p_{j} q_{j}$ and $- 2q_{j}^{2}$ for genotypes *B*_1*j*_ *B*_1*j*_, *B*_1*j*_ *B*_2*j*_ and *B*_2*j*_ *B*_2*j*_, respectively.

Breeding values were predicted with the Bayesian generalized linear regression (BGLR) package [24]. For prediction purposes, variance components were assumed unknown and, thus, estimated simultaneously (marginalized). BGLR implements various Bayesian regression models that were developed for genomic applications, including the GBLUP model. An eigenvalue decomposition of the covariance matrices (${\mathbf{G}}$ and ${\mathbf{D}}$) was used, given its good convergence properties [25]. Default prior parameters and 10,000 iterations plus 2000 burn-in cycles were used in the Markov chain Monte Carlo (MCMC) method, resulting in 100 to 150 effective iterations [26], depending on the parameter and replicate. To verify whether 10,000 iterations were sufficient for our purposes, we compared the prediction of breeding values obtained in chains with 10k and 200k iterations, which was on average equal to 0.98 so we used 10k for computational speed.

The linear predictor included an intercept plus a linear regression on additive effects. We also included both a linear regression on dominant effects and a linear regression on epistatic effects for both E-SEQ and E-QTN evaluation models. Gaussian prior densities were used for all the linear regressions. The residual variance prior was assigned a scaled-inverse Chi-square density and the intercept is assigned a flat prior by default. For the other variances, we also used scaled-inverse Chi-squared densities with hyperparameters set by using the default rules in BGLR (see appendix of [24] at http://www.genetics.org/content/suppl/2014/07/09/genetics.114.164442.DC1/164442SI.pdf). In short, the number of degrees of freedom was 5 (which provides a rather uninformative prior) and scale parameter S such that the R^2^ of the model is matched. BGLR was run at each generation of selection, and predictions were obtained using all the phenotypes and genotypes of all the animals of the preceding generations including the present generation.

### Selection scheme

We generated the base population starting with the 205 sequenced lines of DGRP2 and performing 10 generations of random mating to decrease LD and to generate heterozygous individuals, since parents were homozygous at most sites. The size of the generated base population was N = 500. Selection intensity was 10% in both sexes. At each generation, 25 males and 25 dams were chosen based on genomic breeding values that were predicted using different genomic models (A-SEQ, E-SEQ, A-QTN and E-QTN) and randomly mated; each mating produced 20 offspring with an equal sex ratio. At each generation, breeding values were predicted using all molecular and phenotypic information up to that generation. This scheme was continued for seven discrete generations. Both upward and downward selections were performed. We ran 10 replicates of each of the 16 experiments (two genetic architectures × four evaluation models × two directions of selection). In each replicate, the same set of SNP effects was used, but different base populations with different haplotype structures were generated, although all were initiated with the same real Drosophila data.

### Response to selection, prediction accuracy and additive variance over generations

We investigated the influence of the genetic architecture, the direction of selection and the evaluation model on response to selection, genomic prediction accuracy and the narrow sense heritability over generations. For the total cumulative response to selection, we computed the phenotypic mean per generation (N = 500), averaged across replicates and expressed in standard deviation (SD) units of the base population phenotypic distribution. Prediction accuracy was computed as the Pearson correlation between true and predicted breeding values. Under random mating, the breeding value of an individual is defined as twice the phenotypic mean of its offspring since it deviated from the phenotypic population mean. Calculating true breeding values is straightforward under an additive architecture, since they are equal to the simulated genotypic values. However, this equality does not hold for the epistatic architecture. From the simulations, we have the true total genetic values but not the breeding values. In this case, we used the original definition and empirically estimated the ‘true’ breeding value of an individual that generates 1000 offspring, which result from mating the individual to randomly chosen individuals from the same generation. Additive genetic variance was computed as the variance of ‘true’ breeding values among the individuals in the generation of interest.

### Linkage disequilibrium and inbreeding

Long-range LD between causal SNPs was assessed at the beginning (t_0_) and at the end (t_7_) of each selection experiment and replicate. A pair-wise r^2^ estimation implemented in PLINK [27], defined as the squared correlation coefficient of genotypes at two loci, was used to measure LD between all causal SNP pairs within a chromosome. The curve of the decay of r^2^ with physical distance was fitted for each experiment by nonlinear regression, using Hill and Weir’s [28] expectation of r^2^. Genomic inbreeding coefficient estimates ($F_{\text{h}}$) were obtained for each individual using PLINK’s—het function [27], which is based on excess SNP homozygosity, as $F_{{{\text{h}}_{i} }} = \left( {O_{i} - E} \right)/\left( {k - E} \right)$, where $O_{i}$ is the number of observed homozygous genotype counts for individual $i$, $E = \sum\nolimits_{j = 1}^{k} 1 - 2p_{j} \left( {1 - p_{j} } \right)$ is the number of homozygous genotype counts expected by chance for the base population, $p_{j}$ is the frequency of *B*_1_ in the base population, and $k$ is the number of SNPs.

## Results

**Fig. 1:**
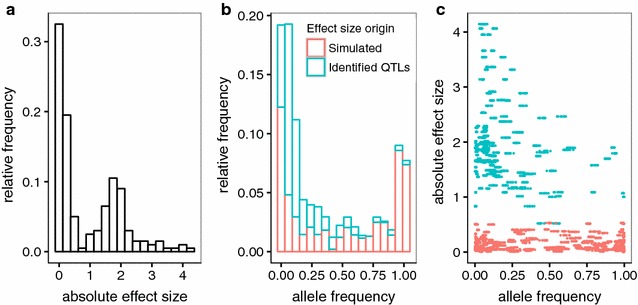
**a** Distribution of absolute additive effects used in the simulations. **b** QTN site frequency spectrum of the allele that increases the trait value in the base population. Sites for which effect size was simulated following an exponential distribution are shown in *red* and those identified in Ober et al. [19] are shown in *blue*. **c** Absolute effect size versus allele frequency of the allele that increases the value of the trait

The distributions of QTN allele frequencies and additive effect sizes in the base population (t_0_) are in Fig. 1. The distribution of absolute effect sizes is bimodal, the result of the empirically identified QTL [19] plus the effects simulated following an exponential distribution (Fig. 1a). The site frequency spectra of the alleles that increase the trait value was U-shaped, although the frequency of alleles that decreased the mean was higher (Fig. 1b). The correlation between effect size and frequency was negative, ρ = −0.42 (Fig. 1c). Note that a negative correlation between effect and allele frequency is expected under some directional or stabilizing selection [17, 29], so our data can mimic a trait that has been under continuous selection.

### Response to selection

**Fig. 2:**
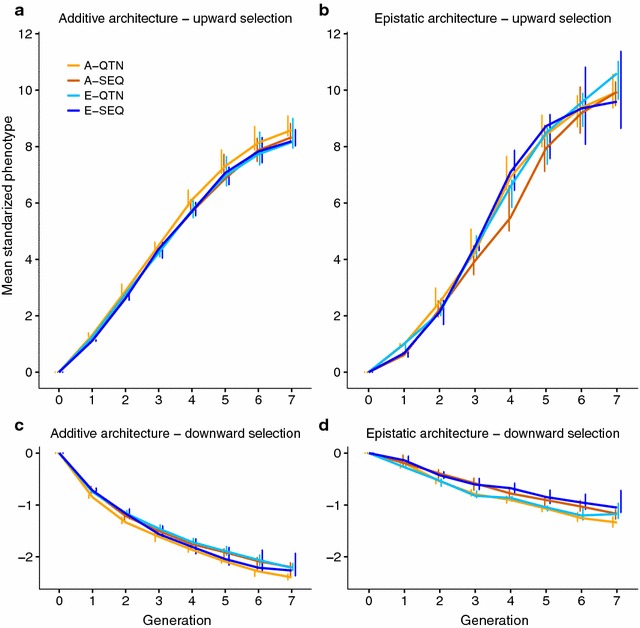
Mode and 75th percentile (*bars*) of the phenotypic mean per generation (N = 500 individuals per generation, 10 replicates). A-QTN: additive model using causal SNPs; A-SEQ: additive model using sequence data; E-QTN: full epistatic model using causal SNPs; E-SEQ: full epistatic model using sequence data

Phenotypic means in SD units along generations and for all scenarios considered are in Fig. 2. Response to selection was clearly asymmetric: for the additive architecture, the upward response was equal to ~8 SD but the downward response was barely 2 SD; epistasis resulted in an even larger asymmetry (10 vs. 1 SD). As discussed below, response was almost exclusively due to changes in allele frequency at loci with a large effect, which were initially at low frequency (Fig. 1b). The frequency of alleles increasing the trait value was 0.17 on average. The complementary model of epistasis actually shrinks the absolute genotypic value of the double homozygotes while it gives the double heterozygote the same advantage as the double dominant homozygote, compared to an additive architecture. In our experiment, epistasis was not symmetrical overall because, for all SNPs, the alternative allele in the Drosophila genome was assigned to be the recessive allele, which happened to be the one that increased the trait value in most cases. Thus, under upward selection, alleles with a positive effect systematically tended to couple (in other words, for double mutants to become favorably selected), which accelerated the rate of response. In contrast, under downward selection, alleles with a positive effect tended to interact negatively on average, which decelerated the rate of response.

The second relevant result is that response to selection depended mainly on the underlying genetic architecture, and not so much on the statistical model used to perform the evaluation (to predict breeding values). For instance, upward response was almost two SD larger in the epistasis architecture than in the additive scenario, irrespective of the evaluation model. Remarkably, knowing the QTN would not have made a big difference. For the additive architecture in particular (Fig. 2a, c), response to selection was rather insensitive to the evaluation model used, even if QTN were known. For instance, upward response using A-QTN was only ~6% larger than that obtained with A-SEQ, E-QTN or E-SEQ. In the presence of epistasis, the situation was somewhat more complex. In this case, knowing the causal QTN improved response to downward selection by about ~24% (A-QTN vs. A-SEQ) or ~37% (E-QTN vs. E-SEQ). In upward selection (Fig. 2b), the advantage of knowing the QTN was only substantial in the short term (~20% higher for t < 5) but was only ~2% onwards. The E-SEQ strategy resulted in an increase in response compared to A-SEQ (on average ~10%) in the medium term and comparable to that with the E-QTN model. Overall, results in Fig. 2b suggest that accounting for epistasis in the evaluation model may have a positive effect if epistasis exists, but mainly in the medium term. Otherwise, in an additive scenario, response is quite insensitive to the evaluation model used to predict breeding values and even to the knowledge of QTN positions.

### Evolution of additive genetic variance and of allele frequencies

**Fig. 3:**
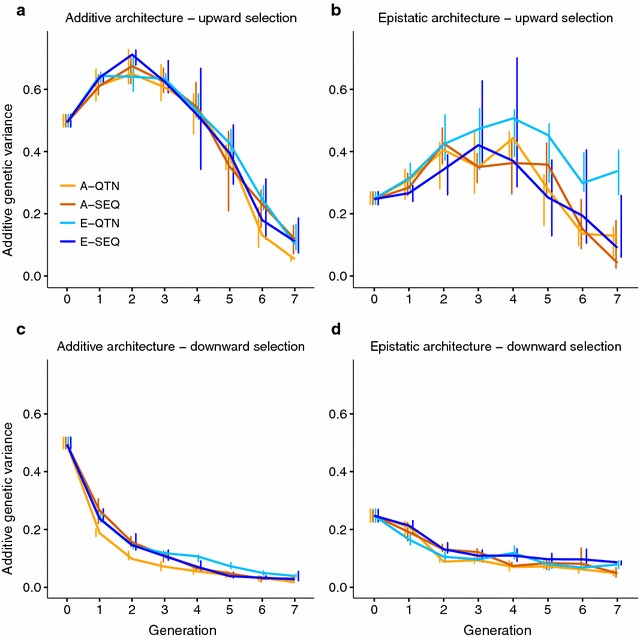
Mode and 75th percentile (*bars*) of the proportion of phenotypic variance due to additive variance (or variance of true breeding values) per generation. A-QTN: additive model using causal SNPs; A-SEQ: additive model using sequence data; E-QTN: full epistatic model using causal SNPs; E-SEQ: full epistatic model using sequence data

The evolution of the additive genetic variance (i.e., the variance of the ‘true’ breeding values, see “Methods”) over generations was examined for each experiment (Fig. 3). Downward selection rapidly eroded additive variance. In contrast, additive variance increased in the first generations of upward selection because alleles with a large effect (Fig. 1b), which were initially at low frequencies, were favored by selection, thus the minor allele frequency increased at those sites. Subsequently, upward selection also exhibited a marked decay in the additive genetic variance. This effect was specially marked in the additive architecture. Importantly, epistasis sustained additive genetic variance for longer, especially if QTN were known and epistasis was included in the model (E-QTN).

**Fig. 4:**
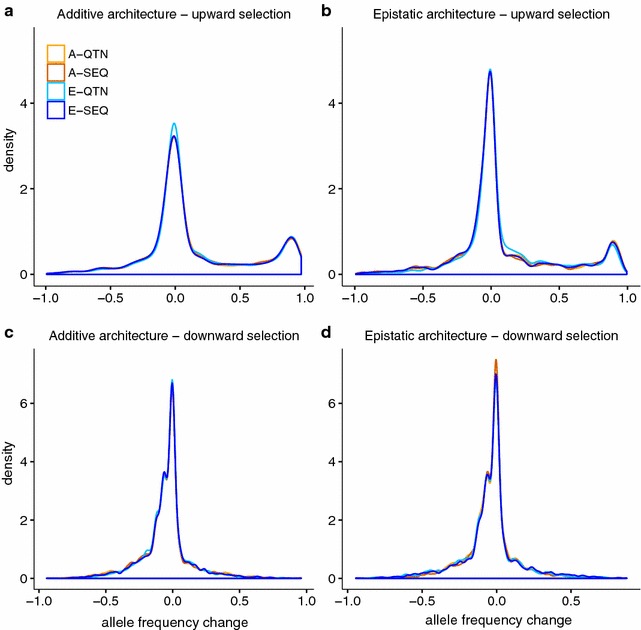
Density plot of total change in allele frequency for the allele that increases the trait value for each of the causal mutations (400 QTN, 10 replicates). A-QTN: additive model using causal SNPs; A-SEQ: additive model using sequence data; E-QTN: full epistatic model using causal SNPs; E-SEQ: full epistatic model using sequence data

For each QTN, we computed the difference in allele frequency between the last and first generation; the distribution of these changes in allele frequencies across the genome is in Fig. 4. Clearly, the evaluation model used for prediction did not have a substantial impact on the change in allele frequency. Yet, the direction of selection and architecture did. First, for downward selection, patterns between additive and epistatic architectures were small. Average changes in allele frequencies were −0.05 and −0.04 for additivity and epistasis, respectively. Again, this is due to the extreme allele frequency that we observed for loci with a large effect in the base population. Second, the pattern of changes in frequency for upward selection was clearly distinct from that for downward selection (Fig. 4a, b). Here, a small percentage of QTN (~7 and 4% for additivity and epistasis, respectively) went to fixation or near fixation starting from very low frequencies, while the frequencies of the other QTN did not change much. In the light of the results in, e.g., Fig. 2a, b, it seems that most of the response was due to these very few loci with a large effect. Note that fewer loci were affected by large changes in frequency with epistasis than with additivity, which again is coherent with the larger decrease in additive variance observed in the additive than in the epistatic architecture (Fig. 4a, b).

To study this furthermore, we computed the expected contribution in the absence of disequilibrium for each locus $j$ (i.e. $2p_{j}$ $q_{j}$ $\alpha_{j}^{2}$, $\alpha$ being the substitution effect [30]). Cumulative distributions both in the base population and in the last generation are in Figure S2 [see Additional file 1: Figure S2]. For all criteria and genetic architectures, selection resulted in fewer loci explaining a given percentage of variance (although note that the absolute value of additive variance decreased as selection proceeded, Fig. 3). About 110 (28%) loci explained 90% of the additive variance in the base population versus 20 to 40 (5 to 10%) loci in the last generation.

### Prediction accuracy

**Fig. 5:**
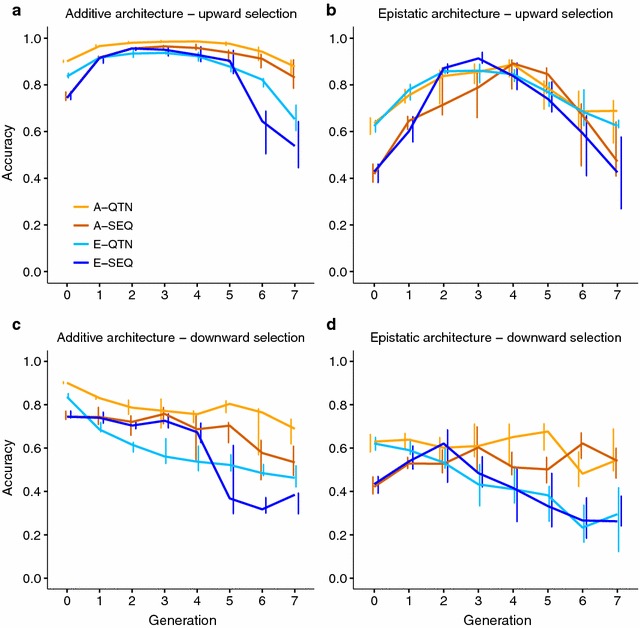
Mode and 75th percentile (*bars*) of the accuracy of prediction of the selection candidates per generation. A-QTN: additive model using causal SNPs; A-SEQ: additive model using sequence data; E-QTN: full epistatic model using causal SNPs; E-SEQ: full epistatic model using sequence data

In contrast to response to selection, prediction accuracy was affected by both genetic architecture and selection method. For the additive architecture and upward selection (Fig. 5a), accuracies obtained with the A-QTN model were high and remained relatively constant over generations; they were only slightly higher than with the A-SEQ model (ca. 4%). If epistasis was absent but accommodated in the model (E-QTN and E-SEQ), accuracies were initially comparable to those of the additive models but decreased eventually. Thus, in the long term, including epistasis in the model when there is none, affected negatively the GS performance, and this was observed in both upward and downward selection (Fig. 5a, c). In contrast, there were marked differences between downward and upward selection accuracies with epistasis (Fig. 5b, d). First, for downward selection, accuracies remained stable with A-QTN and A-SEQ but steadily declined when epistasis was included in the evaluation model. Second, results were clearly non-linear for upward selection. In this case, all accuracies decreased in the long term although there was an advantage of including epistasis in the model in the short term.

### Linkage disequilibrium and inbreeding

**Fig. 6:**
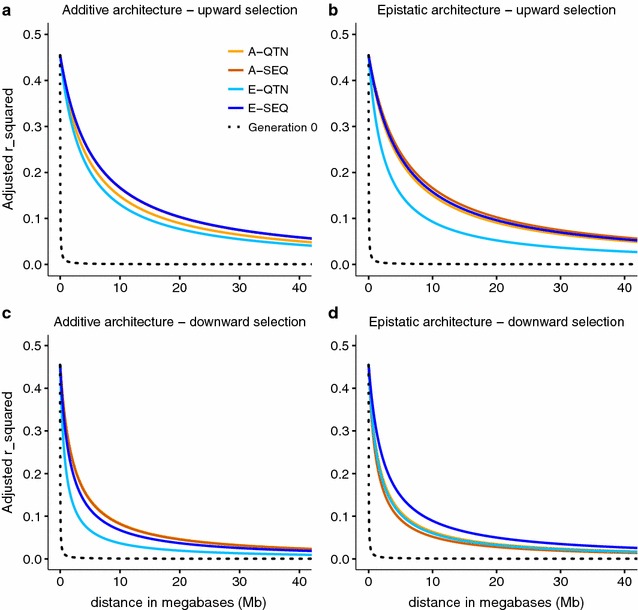
Decay of linkage disequilibrium with physical distance after seven generations of selection. The *dotted line* corresponds to the linkage disequilibrium in the base population. A-QTN: additive model using causal SNPs; A-SEQ: additive model using sequence data; E-QTN: full epistatic model using causal SNPs; E-SEQ: full epistatic model using sequence data

We computed decay of LD (r^2^) with physical distance at the beginning (t_0_) and at the end (t_7_) of each selection experiment. The level of long-range LD in the base population was very low (Fig. 6, black line), with average r^2^ of 0.06 and 0.00 for SNPs within 1 and 5 Mb, respectively. LD increased significantly after selection. Direction of selection seems to be the main factor that affects the extent of LD: upward selection experiments had an average r^2^ of ~0.27 between QTN pairs up to 10 Mb apart, whereas average LD (in r^2^) was ~0.16 for downward selection. This could be due to the fact that most favorable alleles in downward selection were nearly fixed in the initial generation. Genetic architecture did not affect the extent of LD strongly; yet, under the epistatic architecture, knowing the causal mutations and including epistasis in the prediction model hindered the buildup of LD (r^2^ was ~0.21 for E-QTN vs. ~0.28 for the rest of the models).

**Fig. 7:**
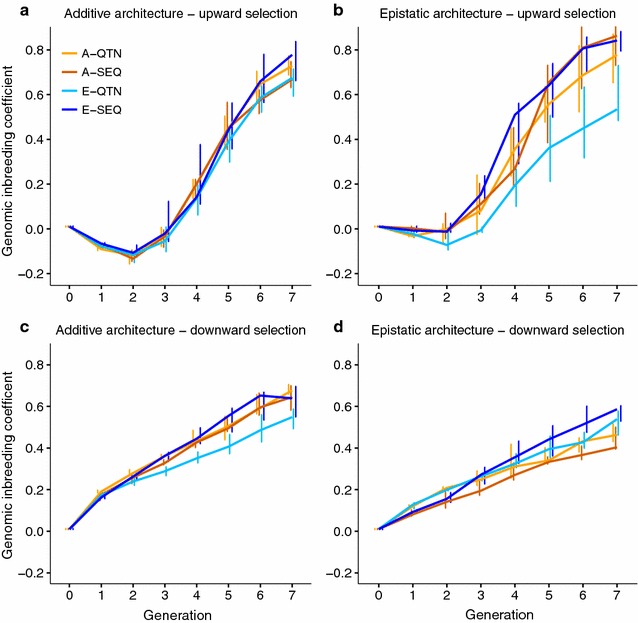
Genomic inbreeding coefficient over generations

There were no substantial differences between genetic architectures in terms of genomic inbreeding over generations, yet the pattern of inbreeding differed with the direction of selection (Fig. 7). After seven generations of upward selection, the final genomic inbreeding coefficient was ~70% on average, compared to ~55% from downward selection experiments. Moreover, an initial reduction of the genomic inbreeding coefficient was observed in upward selection, which is likely related to an initial increase in minor allele frequencies. Knowing the causal mutations can favorably affect genomic inbreeding (on average, 54% with E-QTN versus ~65% for the other evaluation models). Considering that GS is expected to reduce the rates of inbreeding per generation, compared with traditional BLUP, because it provides additional information on Mendelian sampling terms of selection candidates, it is not surprising that the lowest inbreeding corresponded to the model that better reflects the architecture of the trait.

## Discussion

This study examined the impact of epistasis on the short-to-medium-term response to GS in an extreme scenario of non-symmetrical epistasis under divergent selection, as well as the possible advantage of including non-additive effects in the prediction model. To date, there is no previous work on the evolution of selection response to full-sequence-based GS over generations under epistasis.

As in any simulation study of this kind, our study aimed at computational feasibility and made some guesses on a likely genetic architecture. First, although GS is certainly most relevant for mammalian and avian genomes, simulating these large genomes would have added a large computational burden. We chose the Drosophila genome instead because it is about 15 times smaller than those of mammals and because this species has been traditionally used extensively in numerous selection experiments, their chromosome genetic lengths are comparable to those in mammals, and whole-genome sequences are available for a reasonably sized population [17]. Furthermore, starting with a high level of nucleotide variability and a low LD, the intensive selection process that we simulated induced strong LD (Figs. 6, 7), which mimics that of domestic species. Second, we used an extreme epistatic architecture, where all 200 QTN pairs showed interaction. This was done to set an upper limit on the influence of epistasis, for which real effects in comparison to complete additivity are likely weaker than those found here. Third, we used a mixture of estimated and small simulated gene effects. This was done to compensate for the fact that most estimated effects are likely overestimated and result in a negative correlation between effect and frequency, which is expected in traits under directional selection [29].

We used the traditional encoding {0, 1, 2}, where 0 and 2 are for the homozygous genotypes and 1 is for the heterozygous genotype, together with a Hadamard product between ${\mathbf{G}}$ to account for epistasis. It should be recalled that epistasis analyses are coding-dependent [31], because the multiplications of different encodings differ. Recently, we showed that this coding and Hadamard product are equivalent to a model with an explicit effect for interactions [32], and that this model is orthogonal under Hardy–Weinberg conditions. Nevertheless, because we did not build a matrix of dominance-by-dominance or additive-by-dominance epistasis, only part of the dominance is accounted for. Theoretically, more realistic modeling options exist than the one used here but they would require more variance components to be estimated and, in the light of our results, are probably similar to the model used in our study.

In our study, the response to selection was highly asymmetric (Fig. 2) due to the skewed distribution of allele frequencies in the base population. Alleles that decreased the mean were at high frequency in the base population, thus dampening the efficacy of downwards artificial selection [33]. This asymmetric pattern is usually observed in traits that are closely associated with fitness, as is chill coma recovery time [34–37]. The asymmetry was accentuated under epistasis. This can be explained as follows. In the complementary epistatic architecture, our simulated epistasis shrinks the absolute genotypic value of the double homozygotes, compared to an additive architecture (see "Epistatic architecture" in "Methods"). Because the dominant allele happened to be, in most loci, the one that decreased the value of the trait, epistasis is not symmetrical. Under upward selection, the dominant allele will be deleterious and, thus, the double dominant genotype will usually have a fitter phenotype than expected from pure additivity, protecting this positive interaction against the negative effects and causing a less severe fitness drop [38]. In contrast, under downward selection, the dominant allele will usually be the beneficial one and thus the double dominant will usually have a less fit phenotype than expected from an additive action, causing smaller than expected increments in the trait mean. As a result, the rate of response was systematically accelerated (decelerated) under upward (downward) selection, compared to an additive architecture, as predicted by Paixão and Barton [4]. In fact, one of the proposed evolutionary advantages for the presence of epistasis is past selection for resilience to environmental or genetic perturbations [4, 39].

In addition, epistatic interactions affect the response to selection mainly by modulating the additive genetic variance. Here, epistasis sustained additive genetic variance in GS for longer than with pure additivity (Fig. 3b, d). According to Paixão and Barton [4] and Mackay [2], alleles that are initially deleterious or near-neutral may acquire favorable effects as the genetic background changes, “converting” epistatic variance into additive variance, and thus prolonging the response to selection (Fig. 2b, d). In our simulations, downward selection rapidly eroded the additive genetic variance (Fig. 3c, d). This is likely due to the small size of the effective population (although population size was constant and equal to 500 individuals, only 10% in each sex was used to breed the next generation), shift in frequencies of alleles with a large effect, and to the Bulmer effect [40], which induces negative LD (i.e., negative correlation between frequencies of the beneficial alleles) [41–43] and, as a consequence, accuracies also decay rapidly (Fig. 5c, d).

GBLUP genomic evaluation models were robust since adding complexity to the model did not modify substantially the genetic gain for a given architecture. For instance, response was rather insensitive to the evaluation model used in an additive scenario (Fig. 2a, c). Including epistasis when there was none led to similar genomic predictions because the estimated non-additive variance components from GBLUP in this scenario were very close to zero (results not shown). If epistasis existed, including it into the evaluation model had a positive effect, but only in the medium term. This was clearly observed with the E-SEQ model, which achieved response values comparable to those when the causal mutations are known under upward selection (Fig. 2b). Under downward selection and epistasis (Fig. 2d), no substantial effect on the response was observed by adding complexity to the evaluation model, likely because the variation on which selection can work is already very small (Fig. 3d).

Adding complexity to the model had a noticeable impact on prediction accuracy (Fig. 5). For instance, including epistasis in the model when there is none eventually led to lower accuracies as selection proceeded (Fig. 5a, c). Yet, accounting for epistasis when it did exist, did not always lead to higher accuracies; in fact, for upward selection, including epistasis in the model was advantageous only in the medium term when using sequence data compared to using a strictly additive model (Figs. 2b, 5b). This is not too surprising, since most of the genetic variance is additive even in the presence of epistasis, unless allele frequencies are intermediate [2, 5]. (In our scenario of extreme epistasis, narrow sense heritability in the base population was about 0.25, i.e., half the total genetic variance). Accuracies even declined under downward selection when epistasis existed and was included in the evaluation model (Fig. 5d), whereas strictly additive models exhibited relatively constant accuracy values. This may happen because there is not enough power to capture epistatic variance due to most favorable variants being already close to fixation in the base population. Although some studies predict gains in accuracy when accounting for epistasis in GS [19], our results show that this advantage can be eroded as selection proceeds, likely because the substitution effects change over time.

This study also examined the performance of sequence data compared to when the causal mutations are known (i.e., when only the causal SNPs were used to build the genomic relationship matrices). Surprisingly, there was not much gain in response when causal mutations were known (Fig. 2), except at the beginning of the experiment (Fig. 5). With epistasis, the E-QTN model may be beneficial in the long term, because it resulted in significantly lower levels of LD (Fig. 6b) and inbreeding (Fig. 7b) and thus it may sustain additive variance for longer (Fig. 3b). Nevertheless, the small advantage of knowing the causal loci contrasts with previous analyses [19, 44]. In [19], genomic prediction improved when adding SNPs or SNP combinations that were selected based on genome-wide association analyses. In [20], the improvement in prediction accuracy with only-QTN models was larger under an epistatic scenario due to a lower proportion of the total genetic variance being additive in this architecture. However, in our simulations, the initial advantage of knowing the causal mutations on prediction accuracy did not persist. First, this small advantage of knowing the causal mutations may be attributed to the high heritability of the trait, large LD as a result of recent selection and the small number of chromosomes (three in Drosophila), and to the fact that phenotypic information on candidates was available for genomic prediction at each generation.

## Conclusions

This study shows that epistatic interactions affect the response to genomic selection by modulating the additive genetic variance used for selection. Depending on the kind of epistatic action and on the distribution of allele frequencies, epistasis releases additive variance that may increase selection response compared to a pure additive genetic action. Furthermore, genomic evaluation models and, in particular, GBLUP are robust, since adding complexity to the model did not substantially modify the response (for a given architecture). Nevertheless, the evaluation model did affect accuracy but in a nonlinear way, which suggests that complex architectures may require updating the evaluation model as selection proceeds. In practice, of course, the problem would be to have sufficient data to estimate parameters reliably. Finally, even if knowing the causal mutation can in principle boost accuracy, its impact on response to selection can be less impressive than anticipated, especially if the generated LD is very large, the effective population size is small and phenotypes are available on the candidates.

## Additional file

**Additional file 1: Figure S1.** Implementation of selection in SBVB. **(a)** In a given generation *t*, files containing pedigree, phenotypic (Y) and molecular information of individuals up to generation *t* are available, and these are used to perform genomic evaluation of candidates via an external program. **(b)** As a result, estimated breeding values (GEBV) are obtained, and the user selects the sires and dams that will be the parents of generation *t* + 1 (highlighted lines in pedigree file). **(c)** Next, the user needs to expand the pedigree file and generate the pedigree of the next generation, this simply requires adding ids to the pedigree file of as many offspring as desired per each selected couple. In the next round of SBVB, the program will simulate the phenotypes and genotypes (dotted lines) of the new offspring using the parents’ genotypes and the architecture of the trait. **Figure S2.** Cumulative fraction of additive variance across loci. Theoretical individual loci contribution to additive variance, 2*p*(1 − *p*) *α* ^2^, in the base population (black dashed line) and in the last generation for each of the selection criteria and genetic architecture (colored lines). A-QTN: additive model using causal SNPs; A-SEQ: additive model using sequence data; E-QTN: full epistatic model using causal SNPs; E-SEQ: full epistatic model using sequence data.
