# Supplementary material for: Influence of epistasis on response to genomic selection using complete sequence data
Source: Genet Sel Evol. 2017 Aug 25;49:66. doi: 10.1186/s12711-017-0340-3 (PMC5574158; doi:10.1186/s12711-017-0340-3)
Supplement: Supplementary file 1 — Additional file 1: Figure S1. Implementation of selection in SBVB. (a) In a given generation t, files containing pedigree, phenotypic (Y) and molecular information of individuals up to generation t are available, and these are used to perform genomic evaluation of candidates via an external program. (b) As a result, estimated breeding values (GEBV) are obtained, and the user selects the sires and dams that will be the parents of generation t + 1 (highlighted lines in pedigree file). (c) Next, the user needs to expand the pedigree file and generate the pedigree of the next generation, this simply requires adding ids to the pedigree file of as many offspring as desired per each selected couple. In the next round of SBVB, the program will simulate the phenotypes and genotypes (dotted lines) of the new offspring using the parents’ genotypes and the architecture of the trait. Figure S2. Cumulative fraction of additive variance across loci. Theoretical individual loci contribution to additive variance, 2p(1 − p) α 2, in the base population (black dashed line) and in the last generation for each of the selection criteria and genetic architecture (colored lines). A-QTN: additive model using causal SNPs; A-SEQ: additive model using sequence data; E-QTN: full epistatic model using causal SNPs; E-SEQ: full epistatic model using sequence data. [file 12711_2017_340_MOESM1_ESM.docx]

**Figure S1: Implementation of selection in SBVB.** **a)** In a given generation *t*, files containing pedigree, phenotypic (Y) and molecular information of individuals up to generation *t* are available, and these are used to perform genomic evaluation of candidates via an external program. **b)** As a result, estimated breeding values (GEBV) are obtained, and the user selects the sires and dams that will be the parents of generation *t*+1 (highlighted lines in pedigree file). **c)** Next, the user needs to expand the pedigree file and generate the pedigree of the next generation, this simply requires adding ids to the pedigree file of as many offspring as desired per each selected couple. In the next round of SBVB, the program will simulate the phenotypes and genotypes (dotted lines) of the new offspring using the parents’ genotypes and the architecture of the trait.

**Figure S2: Cumulative fraction of additive variance across loci.** Theoretical individual loci contribution to additive variance, 2p(1-p) α^2^, in the base population (black dashed line) and in the last generation for each of the selection criteria and genetic architecture (colored lines). A-QTN: additive model using causal SNPs; A-SEQ: additive model using sequence data; E-QTN: full epistatic model using causal SNPs; E-SEQ: full epistatic model using sequence data.
